# How women's experiences and perceptions of care influence uptake of postnatal care across sub-Saharan Africa: a qualitative systematic review

**DOI:** 10.1186/s12884-021-03910-6

**Published:** 2021-07-13

**Authors:** Caitlin Lythgoe, Kirsty Lowe, Mary McCauley, Hannah McCauley

**Affiliations:** 1 Centre for Maternal and Newborn Health, Liverpool School of Tropical Medicine, Pembroke Place, Liverpool, L3 5QA UK; 2 Liverpool Women’s Hospital, Liverpool Women’s NHS Foundation Trust, Crown Street, Liverpool, L8 7SS UK

**Keywords:** Postnatal care, Respectful care, Perceptions, Experiences, Disrespectful care, Abuse, Pregnancy and childbirth, sub-Saharan Africa

## Abstract

**Background:**

The burden of maternal and neonatal morbidity and mortality is a global health concern with the highest burden documented after childbirth in women and babies living in sub-Saharan Africa. To date, there is limited information on the quality of postnatal care and/or whether evidence-based interventions to improve postnatal care in a way that meets the specific health needs of each mother and her baby have been lacking. There is also limited data related to how quality of care (respectful or disrespectful) influences women's decision to access postnatal care.

**Objective:**

To systematically review available qualitative evidence for how quality of care (respectful or disrespectful) influences perceptions and experiences of, and decisions to, access postnatal care for women living in sub-Saharan Africa.

**Search strategy:**

CINAHL plus, Cochrane library, Global Health, Medline, PubMed, Web of Science were searched from 2009—2019. Grey literature was searched on Google Scholar.

**Selection criteria:**

Qualitative literature in English describing women’s perceptions and experiences of the quality of care they received after childbirth and how this influenced their perceptions of and decisions to access postnatal care.

**Data analysis:**

Thematic analysis was performed to extract subthemes and themes. Outcomes were themes from the qualitative data used to form a thematic synthesis.

**Results:**

Fifteen studies were included with data from 985 women interviewed face-to-face across eight countries. Descriptions of respectful care included healthcare providers being kind, supportive and attentive to women’s needs. Women described preferring healthcare services where the healthcare providers communicated in a respectful and caring manner. Descriptions of disrespectful care included verbal and/or physical abuse and power imbalances between women and healthcare providers. Some women were denied postnatal care when attending a healthcare facility after giving birth at home. There is evidence to suggest that vulnerable women (adolescents; women with poor socioeconomic status; women who are HIV positive) are more likely to receive disrespectful care.

**Conclusions:**

This systematic review describes how aspects of respectful and disrespectful maternity care influence women’s perceptions and experiences of, and decisions to access postnatal care services. There is a need for a renewed focus to prioritise respectful maternity care and to sustainably provide good quality postnatal care to all women and their babies in a way that meets their expectations and health needs.

**Supplementary Information:**

The online version contains supplementary material available at 10.1186/s12884-021-03910-6.

## Tweetable abstract

Adolescent age, poor socioeconomic status, positive HIV status and power imbalances contribute to disrespectful maternity care and act as barriers for women to access postnatal care in sub-Saharan Africa.

## Introduction

The current international aim is to ensure universal access to maternal and newborn health care so that every woman has an equal chance to ‘survive and thrive’ during and after pregnancy [1]. However, this is not the case for many women living in sub-Saharan Africa, where the rates of maternal and neonatal mortality are the highest in the world. An estimated 201, 000 women die every year in sub-Saharan Africa as a result of pregnancy and childbirth, accounting for 66% of the global maternal mortality [2]. Additionally, more than 1 million neonatal deaths occur in sub-Saharan Africa each year, with a baby born in sub-Saharan Africa ten times more likely to die in the first month than a baby born in a high-income country [3, 4].

The majority of maternal health intervention programmes across sub-Saharan Africa have focussed on improving coverage of skilled birth attendance and emergency obstetric and newborn care with the aim to reduce the global burden of maternal deaths, stillbirths and early neonatal deaths, ‘the triple return’ [1]. However, to date, there has been a lack of focus on interventions and programmes that seek to provide comprehensive and individualised care for women and their babies after childbirth. Over half of maternal deaths and the majority of neonatal deaths occur during the first week after childbirth, highlighting that early and good quality postnatal care is crucial to prevent maternal and neonatal mortality worldwide [5, 6]. Postnatal care involves several components of care which both assess the health of the mother and newborn as well as educate the new mother on caring for herself and her child. These evidence-based recommendations are outlined in Table 1.

**Table 1:** Components of postnatal care, adapted from World Health Organization (2013)

**Components of Postnatal Care**
Assessment of the baby
Counselling and support provided for the mother on exclusive breastfeeding
Cord care
Other postnatal care for the newborn (education and recommendations)
Assessment of the mother
Counselling of the mother about the physiological process of recovery after birth and danger signs (includes counselling on birth spacing and family planning)
Iron and folic acid supplementation
Prophylactic antibiotics for women who require them
Psychological support

It is estimated that for every maternal death, 20 to 30 women suffer an acute or chronic morbidity related to pregnancy and/or childbirth [7]. Early detection of ill-health after childbirth as part of routine postnatal care allows for prompt treatment in order to prevent long term maternal and newborn morbidity [8]. Furthermore, good quality postnatal care ensures that newborn vaccinations are completed [9, 10], that women have access to reliable contraception [11, 12], and that women who are HIV positive are supported with measures to prevent mother-to-child transmission of HIV [13].

WHO recommends that all mothers and newborns receive postnatal care within 24 h of childbirth regardless of where the birth takes place, and at least three further postnatal care contacts [14]. However, sub-Saharan Africa has the lowest coverage across all interventions in the reproductive, maternal, newborn and child health continuum of care, with the highest dropout rates reported between skilled birth attendance and postnatal care [14–17]. The increasing recognition of the importance of postnatal care highlights a need for increased coverage [18] but there is concern that the quality of postnatal care must be addressed and prioritised in policy before coverage is increased [19, 20].

The issue of disrespect and abuse during pregnancy and childbirth in sub-Saharan Africa is well documented [21–23], with recognition that disrespect and abuse negatively influence women’s perceptions and experiences of care and that this reduces the likelihood of women delivering at the same facility for future pregnancies [24]. In response to this, the Respectful Maternity Care Charter was published, which outlined the universal rights of childbearing women [25]. The Respectful Maternity Care Charter was updated in 2018 to clearly articulate the rights of the women and newborn during maternity care [26]. The updated charter reflects Agenda 2030 and is directly linked to Sustainable Development Goal 3: ‘Ensure healthy lives and promote well-being for all at all ages’ [27].

Understanding women’s perception of care and experiences of services is important, as perceived quality is an important determinant of service utilization [28]. Women play a central role in assessing and defining quality of care as they choose to access care based on their opinions, and previous experiences with maternal health services [29]. The use of services and outcomes are the result not only of the provision of care but also of women’s experience of that care [30].

There is a need to explore women’s perceptions and experiences to help understand why women stop accessing care soon after childbirth (including cultural and social aspects of care) [31]. Understanding what women consider respectful and disrespectful care to be after childbirth will help to inform patient centred interventions to improve the experience and uptake of postnatal care.

### Objective

To systematically review of qualitative literature for how quality of care (respectful or disrespectful) influences perceptions and experiences of, and decisions to access postnatal care for women living in sub-Saharan Africa.

## Materials and methods

### Data sources and search strategy

Relevant studies published from 1990 to 2019 were identified using a structured search strategy in five electronic databases: CINAHL Plus, PubMed, Global Health, Medline and Cochrane Library. The inclusion of articles published from 1990 to present day was used as this is the same time as the childbirth activism movement began and when studies  started to be published on disrespectful care and abuse and respectful maternity care in maternal health [32]. A search strategy was developed using thesaurus (including MeSH) and free-text terms for “respectful maternity care” “disrespect and abuse” and “postnatal care” with associated keywords were used as main search terms (Supplementary table 1). In addition, the search strategy was applied to Google Scholar to explore grey literature. Reference lists and bibliographies of key studies were searched using Web of Science and any additional studies that met the inclusion criteria were obtained.

### Inclusion and exclusion criteria

Studies were included if they described quality of care (respectful or disrespectful) and perceptions and experiences of postnatal care for women living in sub-Saharan Africa. Studies were excluded if: (i) they were conducted in high income countries; (ii) they reported on healthcare providers’ or mens’ perceptions; (iii) examined women’s perceptions of antenatal or intrapartum care only; (iv) used a quantitative study design only. The review was limited to studies from sub-Saharan Africa as defined by the World Bank. Language was limited to English.

### Selection and data extraction

Two researchers independently screened all titles and abstracts. Evaluation of full-text studies was done independently by two researchers with reasons for exclusion recorded and any discrepancies were discussed with a third researcher.

Information was extracted into a pre-designed summary table and included data on: location of study, study dates, study design, study population, study subject, study outcomes (Supplementary table 2). Throughout the reviewing and extraction processes, studies where uncertainty existed were discussed by all researchers to reach a consensus.

### Quality assessment

The quality of evidence for each study was assessed using the Critical Analysis Skills Programme (CASP) tool (Supplementary table 3) [33]. Studies were reviewed by authors individually. On mutual consensus studies where more than 50% of all

questions on the CASP Tool were satisfied were automatically included for synthesis. Studies that had less than 50% of questions satisfied were discussed and agreement made between the authors regarding inclusion in the final review. In this qualitative systematic review, papers were not excluded based on their quality, with the acknowledgement that the value of papers is often not recognised until the analysis stage [34].

### Data synthesis

The RETREAT framework was used as a guide to select a synthesis method based on the selected studies and a thematic synthesis methodology was chosen [35]. The qualitative results presented in selected studies were coded line by line. This was undertaken in hard copy with codes highlighted manually by the primary researcher (CL). Codes were reviewed by a second researcher (KL) to ensure consistency and allow for discussion of reflexivity. Data that was relevant to the review objectives was extracted. Codes were sorted into higher order codes, subthemes and themes which were reviewed and agreed upon with the second and third researchers (KL, HMC).

## Results

**Fig. 1:**
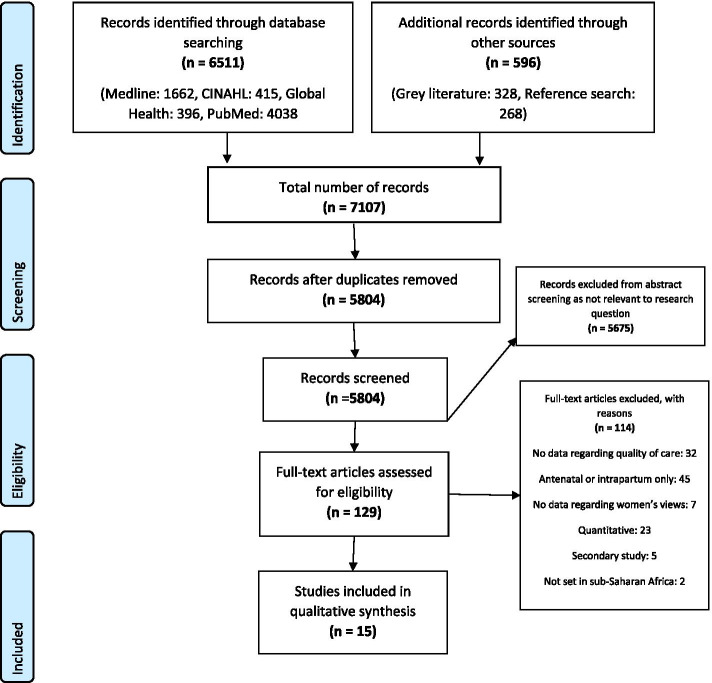
PRISMA diagram outlining the search process

By combining the search terms, 5804 studies were identified and after screening for relevance, 129 were retrieved for full text review (Fig. 1). Upon applying the eligibility criteria, 15 studies met the inclusion criteria. The most common reasons for exclusion were there was no data related to quality of care in the study, antenatal or intrapartum data reported only and the study method was quantitative.

### Characteristics of studies included

#### Geographical spread

Fifteen studies were from eight different countries across sub-Saharan Africa.

Uganda and Zambia had three studies conducted in each country (includes one study conducted in both Uganda and Zambia); Tanzania and Malawi had two studies conducted in each country; and Kenya, Zimbabwe and Ethiopia had one study conducted in each country.

#### Study design, source of data and data collection method

All 15 studies used face-to-face interviews or consultations to collect self-reported primary data from women. Semi-structured interviews, key informant in-depth interviews and focus group discussions were used for data collection. One study supplemented interviews with clinical observation [36]. Eight of the 15 studies assessed women after childbirth only and seven studies explored women’s views on postnatal care as part of the continuum of maternal care. Thematic analysis was used in all but two of the studies [37, 38] both of which used a thematic framework analysis. Although different methodologies, both analysis methods are descriptive in nature making the combining of results feasible.

#### Study population

985 women were interviewed across the 15 included studies. Seven of the 15 studies included the perceptions of healthcare providers and male participants in addition to women’s own perceptions and experiences. For these studies, only data from women who had given birth were extracted.

#### Setting and site of data collection

Two studies were conducted in urban settings [37, 39]; nine in rural settings [36, 38, 40–46]; and four studies were in both urban and rural settings [47–50]. Interviews were conducted with women in a variety of settings: their homes [40, 49], community centre [40], 'private locations' [41, 43, 47, 48, 50], rural healthcare centre [36], village based informant’s house [42], maternity unit at hospital [37], research site [49], at the bedside in the postnatal ward [49] and in some studies the interview site was unclear [38, 39, 41, 44, 46].

### Qualitative results

The themes identified are summarised across two areas 1) respectful 2) disrespectful care and by sub-themes. The subthemes related to respectful care are; positive experiences of respectful care, women-centred care, respectful and kind healthcare providers  providing care, care where women’s needs are prioritised, and informed choice. The subthemes identified regarding disrespectful care are: stigma and discrimination, non-dignified or disrespectful care, physical abuses and denial of care. Content quotes are provided in Tables 2 and 3.

**Table 2:** Descriptions of respectful postnatal care

**Respectful maternity care**		
**Themes**	**Sub themes**	**Content quotes**
**Positive experiences of respectful care**	Women value respectful and kind treatment [36, 37, 39, 42, 48, 49]	“We would want an audience with the District Nursing Officer to tell her that she should recruit people who have the love to serve everyone with respect.” – [36] “I feel like [the trainees] are the ones who work with [more] commitment than those … qualified [nurses]. These trainees they are not experienced … but if I [could] choose who [should help me], I would go for a trainee … because he or she is going to be fair … It’s not about being qualified, it’s about the … kind of services.” [37]
	Choosing to access services where healthcare workers are respectful [37, 48]	“those doctors, those who treat us at X [name of community/clinic] [are friendly], which is why when another day they told me they would give transfer to Y [name of community/clinic] I told them I would rather pay that fare [higher fare to go to X clinic] and go to X [name of community/clinic]. They are friendly, and they give you the chance to ask the questions that you want. There you are free.”[48]
**Women-centred care**	Women feeling their needs are prioritised [40, 45]	“While I was giving birth [at home], the placenta could not come out safely, but when I went to the hospital, I was helped by a welcoming doctor who left all what he was doing to come and attend to me.” – [49, 45]
	Support from health care providers [49]	“I just feel that we need support from them. If the nurse is supportive, then that's all I need her to have.” – [49]
	Informed choice [37, 44, 48, 49]	“I would not feel discriminated if I do not breastfeed my baby… because we were attending the teachings at the MCH [mother and child health] clinics, they [the nurses] emphasized to us that ‘this time, it is your responsibility, you can choose for yourself the feeding method you want to use to raise your baby with’…these are the things I can tell others [members of community who question her breastfeeding practices]’.” – [48]
	Communication [37, 42, 45]	“… it’s best that there’s actually communication… [so] that you can actually tell them everything that you’re experiencing [and] they can actually help you.”[37]

**Table 3:** Disrespectful postnatal care

**Disrespectful maternity care**		
**Themes**	**Sub themes**	**Content quotes**
**Stigma and discrimination based on specific attributes**	Birth-place related stigma [40, 43, 45, 47]	“They tell us to come back when we can explain ourselves to see if they are satisfied with the explanation [for having delivered at home].” – [45] “If a woman gives birth at home, the HEWs [health extensions workers] won’t hear about it. Nobody tells them that she has given birth” – [40]
	Stigma related to HIV status [39, 46, 50]	“When you get there, they are shouting at you as if you are a small child, that why I think people stop going to clinics.” – [39]
	Age related discrimination [36, 40, 49]	“They were just talking, saying I am so uncooperative and yet I am so young and why am I doing all these things if I am not going to be able to face up to it” – [49]
	Discrimination due to socio-economic status [36, 50]	"At [public hospital], they [nursing staff] were shouting. They say you foreigner, come to make a baby here." – [50] “…one would never go wrong with the midwives if they came for their check-ups well groomed” – [36]
**Non-dignified and disrespectful care**	Negative attitude of health care providers [36, 42, 43, 49]	"When we go late to the clinic for postnatal care, the healthcare providers complain that they are getting a meagre salary and yet we keep bothering them" – [42] “Attitude, the way sometimes patients get treated, it’s not OK for me…sometimes you find that people are afraid to ask certain things because you find that health workers can’t be approached …it’s not OK the way that they deal with patients. I am not happy.”—[39]
	Power imbalance [36–38, 45]	“Some of the midwives are not approachable. If you try to exercise your rights it appears as if you are going too forward ahead of the midwives” – [36]
	Hostile environment [[36, 38]]	“Many pregnant mothers are waiting. When you leave, another woman gets the bed. Our wards are few and we are many. Like if you were to continue staying you would bring trouble for others… and the nurses tell us, ‘Now the time has come. You should go so that others can come.’” – [38]
**Abuse**	Physical abuse [39, 43, 45–47]	“No we did not go to the hospital [for postnatal care] because if they notice that you delivered from the village … her [the nurse] can easily beat you. So it is better I go to the old lady in the village because I know she will treat me well.” – [45]
**Denial of care**	Denial of postnatal care after delivering [38, 40, 42, 45, 47]	“Stay longer … if you have delivered safely you can’t stay. The only time they say you stay for 24 h is if you have problems. We know the rules.” –[38]
	Denial of care as a punitive measure [40, 45, 47]	“Me, after delivering I spent 1 week when I had no problem; even the baby was suckling and sleeping well. After 3 days she got fever and she was really badly off. I said let me take her to the facility, maybe I would be helped. They told me the medicine is not there for me [because I had delivered at home].” – [45] “If you go to the clinic after giving birth at home, nurses make you pay before they examine your baby” – [47] “I didn’t go [for postnatal care] because I didn’t have ZMW51 to pay for the card.”- [45]
	Lack of informed care [36, 38, 39, 42, 43, 45, 49]	“I do not have much understanding of the programme [maternal health services]. I am a first-time mother, so I don't know about many things. Neither do the midwives see the needs to tell me anything.” – [36] “No. They’re [new mothers] not supposed to go to the clinic if the baby is OK and they are OK. I don’t see the point of me always being at the clinic. What will I be doing there? Because the baby is healthy and I am healthy.” – [39]

#### Respectful care

In many studies women valued respectful care and expressed a desire to be treated with respect and kindness by healthcare providers [36, 37, 39, 42, 48, 49]. In several studies, being respectful was identified as the most important attribute for a healthcare provider to have [36, 42]. Women explained that they valued how they were treated by the healthcare providers more than the level of clinical experience that the healthcare providers had [37, 48].

Women described respectful care after childbirth as when healthcare providers displayed a positive attitude towards them [41, 45] and were attentive and sensitive to their needs [40, 45]. Women valued information and education as part of postnatal care [37, 44, 48, 49]. Women wanted to be able to make informed decisions about their care after childbirth [37] and they expected healthcare providers to give them all the necessary information to be able to do so [42, 45]. Women valued the healthcare providers’ opinion and trusted their judgement [42].

#### Disrespectful care

Across the included studies generally more examples of disrespectful care than respectful care was provided.

#### Verbal and/or physical abuse

Interactions with healthcare providers influenced women’s decisions to use postnatal care; with some women choosing not to access postnatal care due to fear of mistreatment from healthcare providers, including physical and verbal abuse [39, 43, 45–47]. Although some of these fears had originated from the accounts of other women, most women feared healthcare providers due to previous experiences they themselves had encountered throughout their antenatal or labour care [45, 47, 50]. Some women described that they were shouted at by the healthcare providers in front of other mothers at the clinic, which caused them to feel humiliated.

#### Healthcare providers’ negative attitudes

Women described experiences of disrespectful care with healthcare providers who had negative attitudes, describing them as unfriendly and in some cases threatening [36, 42, 43, 49]. Negative attitudes from healthcare providers resulted in women being too afraid to ask questions, finding healthcare providers ‘unapproachable’ and fearing their response [36, 39]. By avoiding asking questions some women did not have access to enough information to be able to properly consent to treatment [36].

Some women reported not understanding the importance of postnatal care due to a lack of information from healthcare providers [36, 42]. This meant that women were unsure of the postnatal care programme when questioned about it.

This lack of education about the importance of postnatal care was reflected in women’s knowledge of postnatal care, with women not understanding the need to attend postnatal care if both her and her child were well [38, 43, 45, 49]. Some of these women had been told by healthcare providers not to come back unless they had a medical problem after delivery.

The power imbalance between healthcare providers and women was highlighted in four studies [36–38, 45], with many women describing how the healthcare providers made decisions on their behalf and that women feared that they would not receive care if they did not allow the healthcare providers to make these decisions for them. Some women felt that they would be perceived as ‘insulting’ the authority of the healthcare providers if they asked questions [38].

Some women described experiencing an ‘uncomfortable environment’ during childbirth and this discouraged them from coming back to a healthcare facility for postnatal care services [36, 38]. Women described being encouraged to leave the facility straight after birth by healthcare providers which they perceived as being due to the lack of beds and adequate space for them to remain for at least the recommended minimum of 24 h after birth [38]. Some women accepted disrespectful care as ‘normal’ or justified it due to the stressful job of the midwife [36].

#### Stigma and discrimination

Examples of stigma and discrimination were reported by women in the majority (12/15) of studies. This included discrimination due to age, socio-economic status, positive HIV status.

Adolescent mothers described how healthcare providers would belittle them in front of other healthcare providers [49], and how they would not properly explain things in a way that would enable them to make informed choices [36]. This resulted in young women keeping their pregnancies and births secret and not accessing postnatal care for themselves or their babies [40]. Women reported stigma related to a woman’s nationality [50] and socioeconomic status, with women ensuring they appeared ‘well-groomed’ for postnatal clinic appointments to avoid stigma [36].

Women who were HIV positive reported discrimination from healthcare providers due to their HIV status describing verbal abuse [39, 50] and being humiliated in front of other mothers in the postnatal clinic [50]. Additionally, women living with HIV felt that they were treated differently by healthcare providers in a negative way during their postnatal appointments, compared to women who were not HIV positive [46]. Women who experienced this discrimination described not wanting to attend postnatal appointments due to the stigma they faced [39, 50]. In the included studies there were multiple examples of women explaining that they would access more care if they were treated better by the healthcare providers [36, 37, 39, 40, 42, 48, 49].

#### Denial of care

Women described being denied postnatal care either in the healthcare facilities or at home in five of the included 15 studies [38, 40, 42, 45, 47]. Women reported being told to leave the healthcare facility after birth by healthcare providers before the recommended 24 h, despite some women wanting to stay for longer [38, 42].

Women were also refused postnatal care after a home delivery, as a punishment for not delivering in the facility [40, 45]. Women described being chastised by healthcare providers for having had a planned home birth. Women reported that healthcare providers would not attend home visits for these women and when they attempted to access the facility for postnatal care they were turned away and denied medication, being told that they were a lower priority to women who had delivered in the facility [40, 43, 45, 47].

Women were also denied care unless they paid unofficial ‘under the table’ fees to healthcare providers [45, 47]. These unofficial payments were requested from women who had delivered at home; with women turned away due to their inability to pay for health cards or examinations which would normally be provided free of charge.

## Discussion

### Main findings

This systematic review explores the perceptions and experiences of 985 women living in sub-Saharan Africa and illustrates that women have a clear understanding of what respectful care looks like—describing this as healthcare providers being kind, attentive to women’s needs, and supportive. When women received respectful care, they had a positive perception and experience of postnatal care and reported being more likely to access care for subsequent health needs in settings where healthcare providers communicated in an empathic and caring manner.

Women reported power imbalances between themselves and the healthcare providers responsible for their care. Women described feeling afraid of healthcare providers due to previous experiences of physical and/or verbal abuse and described disrespectful care such as being denied postnatal care if they had delivered their baby at home. Some women felt that they had no choice but to follow the demands of healthcare providers or face denial of care. Studies document that vulnerable women (adolescents; women with poor socioeconomic status; women who are HIV positive) are more likely to report having received and experienced disrespectful care from healthcare providers.

### Strengths and limitations

To the best of our knowledge, this is the one of the first systematic reviews to summarise the evidence that reports women’s perceptions and experiences of respectful and disrespectful maternity care after childbirth and how this influences women’s perceptions of and decision to access postnatal care across sub-Saharan African countries. By using a comprehensive search strategy, we captured descriptions of women’s perceptions of postnatal care within current literature. There were common themes and subthemes reported by women in many of the studies suggesting women’s experiences are similar across different contexts and countries. Few studies explored what respectful care looks like in the specific contexts especially with regard to cultural and societal expectations of women with newborn babies, highlighting the need to prioritise women’s needs and voices in future research. None of the studies included women who have suffered a stillbirth.

### Interpretation

Data surrounding women’s perceptions and experiences of and decisions to access postnatal care to date are limited in sub-Saharan African countries, which confirm the need for a new approach and focus. A review of studies illustrates that women value respectful care as a priority when making decisions about accessing postnatal care. Similar findings have been described in studies exploring care during pregnancy and childbirth [28, 51, 52]. Being treated with respect, receiving support and good communication from healthcare providers are all valued by women during pregnancy and childbirth, and are predictors of women’s satisfaction with maternal health services [28, 51, 52].

Disrespectful care during childbirth has been well documented [53] and is a known barrier to women choosing to give birth in healthcare facilities [54]. However, there is less evidence about the significance of disrespect and abuse in the postnatal period. We found that women’s negative experiences during antenatal care and childbirth, or during postnatal care in previous pregnancies, prevented them from seeking postnatal care.

Stigma and discrimination were underlying issues across the themes. Stigma attached to women who chose to have a home birth may be due to the current international agenda recommending that all women give birth with a skilled birth attendant at healthcare facilities [27, 55]. Several African countries have made this mandatory and some healthcare facilities have introduced unofficial financial penalties if women do not access care at time of birth in a healthcare facility. Global health initiatives which focus on the quantity of facility deliveries, often associated with funding, put pressure on local health implementers and can create unhealthy incentives such as sanctioning women for delivering at home with fines [56]. This in turn can prevent women accessing postnatal care due to fear of healthcare providers and not being able to afford unofficial sanctions, unintentionally widening inequity and impacting the quality of care [57].

Denial of care is well documented as an abuse of women during childbirth, but there is limited literature on the denial of postnatal care across sub-Saharan African countries. In our systematic review, women were apprehensive about seeking postnatal care if they had delivered at home through fear of the negative reactions from healthcare providers and being denied care. This may play a significant role in women’s negative perceptions of postnatal care.

In our systematic review adolescent mothers describe that healthcare providers did not respond to their needs and that they felt judged. The treatment of pregnant adolescents in sub-Saharan Africa is recognised as an issue, with adolescents facing more challenges during their pregnancy due to community stigma and violence, meaning this group is less likely to seek care [58]. As a consequence, adolescent mothers have a higher risk for pregnancy complications [59]. It is recommended that healthcare services are adolescent friendly in order to provide equitable care for adolescents [60].

Women’s experience of discrimination due to their HIV status negatively influenced their perception of postnatal care and discouraged utilisation. Pregnant and postpartum women are a vulnerable group in relation to HIV stigma, as they are often the first in their families to be tested for HIV they can be seen as the person with whom the disease originates [61]. Additionally, with the integration of HIV and maternal health services, seeking care can be associated with unwanted disclosure and stigma [61]. The stigma and discrimination faced by women has been shown to have a negative impact on all stages of the ‘Prevention of Mother to Child Transmission of HIV’ cascade and has a significant effect on service uptake and adherence [61]. Further assessment of HIV related stigma during postnatal care is needed in order to inform ‘Prevention of Mother to Child Transmission of HIV’ programmes in sub-Saharan Africa.

Disrespectful care creates fear of healthcare providers and leads to power imbalances between women and healthcare providers. In our review, women felt that they had no choice but to follow the demands of healthcare providers or face denial of care. There have been many theoretical discussions about the driving forces behind disrespectful care and how power dynamics play their part in disrespect and abuse. These studies propose that if there is a background of organisational issues and disempowering environments, midwives attempt to exert power over women in an attempt to secure their own status in a hierarchical workplace [62–64].

## Conclusion

 Few studies to date have focussed on women’s perceptions and experiences of care after childbirth, and the results of this systematic review provide ample evidence and examples for the urgent need to address aspects of care that are disrespectful and that are likely to contribute significantly to the current low uptake of postnatal care across sub-Saharan Africa.

With a refocus from coverage to quality of care during pregnancy and childbirth, this review highlights the need for respectful care for women after childbirth also. We recommend that all postnatal care interventions implement respectful care principles from the design stage and comprehensive and routine auditing of respectful care standards during postnatal care is introduced to inform policy and program decisions and for resource allocation for postnatal care.

## Supplementary Information

**Additional file 1: Supplementary Table 1.** MEsH terms. **Supplementary Table 2.** Summary of included studies. **Supplementary Table 3.** Critical Skills Appraisal Programme (2015) summary table.

## Data Availability

All the sources of data are publicly available and referenced in the document. The datasets analysed during the current study are available in the CINAHL Plus [https://www.ebsco.com/products/research-databases/cinahl-plus], PubMed [https://pubmed.ncbi.nlm.nih.gov/], Global Health [https://www.ebsco.com/products/research-databases/global-health], Medline [https://www.nlm.nih.gov/medline/index.html] and Cochrane Library [https://www.cochranelibrary.com/] repository.

## Abbreviations

CASP: Critical Analysis Skills Programme
GRADE: Grading of Recommendations, Assessment, Development and Evaluation
LMIC: Low- and middle-income countries
MeSH: Medical Subject Headings
PRISMA: Preferred Reporting Items for Systematic Reviews and Meta-Analyses
RETREAT: Review question, Epistemology, Time/Timescale, Resources, Expertise, Audience and purpose, Type of Data
WHO: World Health Organization
