# Supplementary material for: How women's experiences and perceptions of care influence uptake of postnatal care across sub-Saharan Africa: a qualitative systematic review
Source: BMC Pregnancy Childbirth. 2021 Jul 13;21:506. doi: 10.1186/s12884-021-03910-6 (PMC8276494; doi:10.1186/s12884-021-03910-6)
Supplement: Supplementary file 1 — Additional file 1: Supplementary Table 1. MEsH terms. Supplementary Table 2. Summary of included studies. Supplementary Table 3. Critical Skills Appraisal Programme (2015) summary table. [file 12884_2021_3910_MOESM1_ESM.docx]

**How experiences and perceptions of care influence uptake of postnatal care across sub-Saharan Africa. A qualitative systematic review.**

Lythgoe, Caitlin^1^

Lowe Kirsty^1^,

McCauley, Mary^1,2^

McCauley Hannah^1^*

^1^Centre for Maternal and Newborn Health, Liverpool School of Tropical Medicine, United Kingdom

^2^Liverpool Women’s Hospital, Liverpool Women's NHS Foundation Trust, Crown Street, Liverpool, United Kingdom

*Corresponding author:

Hannah McCauley

Centre for Maternal and Newborn Health

Liverpool School of Tropical Medicine

Pembroke Place

Liverpool L3 5QA

United Kingdom

Email:Hannah.McCauley@lstmed.ac.uk

**Supplementary Table 1: MEsH terms**

| **Term** | **MEsH Terms** |
| --- | --- |
| **Postnatal** | (MH “Postnatal Care”) OR (MH “Postpartum Care (Saba CCC)”) OR (MH “Postnatal Period”) OR (MH ”Puerperium”) OR “postnatal care” OR “postnatal” OR “after birth” OR “postpartum” OR “puerperium” OR “post-pregnancy” OR “post-birth” |
| **Sub-Saharan Africa** | “Africa” OR “SSA” OR “sub Saharan Africa*” OR “Angola” OR (MH “Angola”) “Gabon” OR (MH “Gabon”) OR “Nigeria” OR (MH ”Nigeria”) OR “Benin” OR (MH “Benin”) OR “Gambia” OR (MH “Gambia”) OR “Rwanda” OR (MH “Rwanda”) OR “Botswana” OR (MH “Botswana”) OR “Ghana” OR (MH “Ghana”) OR “São Tomé and Principe” OR (MH “São Tomé and Principe”) OR “Burkina Faso” OR (MH “Burkina Faso”) OR “Guinea” OR (MH “Guinea”) OR “Senegal” OR (MH “Senegal”) OR “Burundi” OR (MH “Burundi”) OR “Guinea-Bissau” OR (MH “Guinea-Bissau”) OR “Seychelles” OR (MH “Seychelles”) OR “Cabo Verde” OR (MH “Cabo Verde”) OR “Kenya” OR (MH “Kenya”) OR “Sierra Leone” OR (MH “Sierra Leone”) OR “Cameroon” OR (MH “Cameroon”) OR “Lesotho” OR (MH “Lesotho”) OR “Somalia” OR (MH “Somalia”) OR “Central African Republic” OR (MH “Central African Republic”) OR “Liberia” OR (MH “Liberia) OR “South Africa” OR (MH “South Africa”) OR “Chad” OR (MH “Chad”) OR “Madagascar” OR (MH “Madagascar”) OR “South Sudan” OR (MH “South Sudan”) OR “Comoros” OR (MH “Comoros”) OR “Malawi” OR (MH “Malawi”) OR “Sudan” OR (MH “Sudan”) OR “Democratic Republic of Congo” OR (MH “Democratic Republic of Congo”) OR “Mali” OR (MH “Mali) OR “Swaziland” OR (MH “Swaziland”) OR “Republic of Congo” OR (MH “Republic of Congo”) OR “Mauritania” OR (MH “Mauritania”) OR “Tanzania” OR (MH “Tanzania”) OR “Côte d'Ivoire” OR (MH “Cote d’Ivoire”) OR “Mauritius” OR (MH “Mauritius”) OR “Togo” OR (MH “Togo”) OR “Equatorial Guinea” OR (MH “Equatorial Guinea”) OR “Mozambique” OR (MH “Mozambique”) OR “Uganda” OR (MH “Uganda”) OR “Eritrea” OR (MH “Eritrea”) OR “Namibia” OR (MH “Namibia”) OR “Zambia” OR (MH “Zambia”) OR “Ethiopia” OR (MH “Ethiopia”) OR “Niger” OR (MH “Niger”) OR “Zimbabwe” OR (MH “Zimbabwe”) |
| **Respectful maternity care and disrespect and abuse** | “respectful maternity care” OR “wom#n cent#red care” OR “patient cent#red care” OR (MH “Quality of Health Care”) OR “quality of care” OR (MH “Patient Centered Care”) OR “midwifery standard*” OR “medical care standard*” OR “interpersonal relation*” OR “attitude*” OR “abus*” OR “disrespect*” OR “disrespectful maternity care” OR “discriminat*” OR “stigma” OR “dignified care” (MH “Patient Abuse”) OR (MH “Quality of Nursing Care”) OR (MH “Respect”) OR (MH “Cultural Competence”) OR (MH “human dignity”) OR (MH “Nurse-Patient Relations) OR (MH Midwife attitudes) OR (MH “attitude of health personnel”) |

**Supplementary Table 2: Summary of included studies**

| Study | Country | Setting | Interview setting | Aim | Area of interest | Participants | Methodology |
| --- | --- | --- | --- | --- | --- | --- | --- |
| Early postnatal home visits: a qualitative study of barriers and facilitators to achieving high coverage (Amare *et al*, 2018) | Ethiopia | 4 districts with predominantly subsistence farming based economy | Respondent’s houses and community centres | To explore the barriers and facilitators to early postnatal visits by community health extension workers | Postnatal care | Recent mothers, grandmothers, fathers, health extension workers, health development army within the 4 districts | Narrative interviews (12), in-depth interviews (13), friendship pair interviews (5), FGD’s (6). Thematic analysis |
| Postnatal Care Experiences and Barriers to Care Utilization for Home- and Facility-Delivered Newborns in Uganda and Zambia (Sacks *et al*., 2017) | Uganda and Zambia | 8 rural districts | Private locations | To examine experiences with and barriers to, accessing postnatal care services, in the context of a maternal health initiative | Postnatal care | Women who had delivered in the preceding year and lived within one of the 8 districts | Focus group discussions (48). Thematic analysis |
| Women’s experiences of disrespectful and abusive maternal health care in a low resource rural setting in eastern Zimbabwe (Kanengoni *et al,*. 2019) | Zimbabwe | 3 rural health centres within a low resource setting | 3 rural health centres | To explore women’s experiences and perceptions of disrespect and abuse from their maternity care providers in a low resource rural setting in Zimbabwe | Antenatal care, postpartum care and maternity waiting homes’ services | Women 16-30 years of age who were accessing various stages of maternal health services, recruited from 3 selected health centres. | In depth interviews (8), focus group discussions (2 focus groups: 8 women in first focus group, 4 in second focus group), observation at clinics. Thematic analysis |
| “You should go so that others can come”; the role of facilities in determining an early departure after childbirth in Morogoro Region, Tanzania (McMahon et al., 2015) | Tanzania | 4 districts within a rural region | Unclear | To present findings on time to discharge, characteristics of women who depart early, receipt of postpartum messaging and the experience of post-delivery facility departure as described by women in rural Tanzania | Postnatal care in facility after childbirth | Women who had delivered in a facility in the past 14 months, their husbands and community leaders within the 4 districts. | Mixed methods study. In depth interviews (41). Qualitative data analysed using framework approach. |
| The use of antenatal and postnatal care: perspectives and experiences of women and health care providers in rural southern Tanzania (Mrisho et al., 2009) | Tanzania | 8 rural villages | Village based informant’s home | To describe the perspectives and experiences of women and health care providers with regard to use of ANC and postnatal care in order to identify opportunities for improving maternal and newborn health services | Antenatal and postnatal care | Village based informants (8), Health Care Providers (8) and women with babies ages less than one year of age (39), pregnant women (19) | Data collected as part of larger project. In-depth interviews for the health care providers and village based informants (16). Focus group discussions for women (8). Thematic analysis. |
| Use of postpartum health services in rural Uganda: knowledge, attitudes, and barriers (Nabukera et al., 2006) | Uganda | 2 rural communities | Private places | To explore the knowledge, attitudes and barriers to use of postpartum care service among rural communities in Uganda | Postpartum care | Local political leaders (5), opinion leaders (5), women representatives (5), health care providers (5), religious leaders (5), in school and out of school youth (5), peer educators (5), traditional birth attendants (5), teachers (5), ordinary persons from the community (5). | Qualitative report on a mixed methods project. Narrative enquiry method. One-to-one interviews (50). Thematic analysis. |
| Understanding the psychosocial and environmental factors and barriers affecting utilization of maternal healthcare services in Kalomo, Zambia: a qualitative study (Sialubanje et al., 2014) | Zambia | Mixed urban and rural health centre catchment areas | A quiet place in each village | To identify psychosocial and environmental factors and barriers affecting utilization of maternal healthcare services in Kalomo, Zambia | Antenatal care, delivery services and postnatal care | Women of reproductive age who gave birth within the last year (141) , traditional leaders, mothers, fathers, community health workers and nurse-midwives (35) | Focus group discussions (12), In-depth interviews (35). Data analysed according to pre-existing themes. |
| “I just wish that everything is in one place”: Facilitators and barriers to continuity of care among HIV-positive postpartum women with a non-communicable disease in South Africa (Clouse et al., 2018) | South Africa | Hospital within an urban city | Unclear | To explore healthcare utilization among postpartum women requiring chronic management of HIV and NCD, and identify facilitators and barriers to follow-up engagement | Postpartum care | Women over 18 years old who are HIV positive, recently postpartum, and diagnosed with a non-communicable disease that required further evaluation after pregnancy | One-to-one open ended interviews, a conceptual model was developed from the themes. |
| The role of the parents’ perception of the postpartum period and knowledge of maternal mortality in uptake in postnatal care: a qualitative exploration in Malawi (Zamawe et al., 2015) | Malawi | District which is mainly rural- community setting | Varied- places and times chosen by participants | To examine the perceptions of parents towards the postpartum period and postnatal care in order to deepen our understanding of the maternal care-seeking practices after childbirth | Postnatal care | Parents aged 18-35 years who had either given birth or fathered a baby within 12 months prior to the study | Descriptive qualitative study, focus group discussions (4). Thematic analysis |
| “We are the ones who should make the decision”- knowledge and understanding of the rights based approach to maternity care among women and healthcare providers (Jolly et al., 2019) | Malawi | Large referral hospital in a city in Malawi (urban) | Maternity unit of large referral hospital | To explore knowledge and understanding of the seven domains of the respectful maternity care charter among healthcare providers and to explore women’s perceptions regarding respectful maternity care | Antenatal care, delivery services, postnatal care | Mother attending antenatal clinic, mothers attending postnatal clinic, women accessing intrapartum services, healthcare providers | Focus group discussions (8) for women attending ANC and postnatal care, in-depth interviews (9) with healthcare providers. Thematic framework analysis |
| Experiences of stigma among women living with HIV attending sexual and reproductive health services in Kenya: a qualitative study (Colombini et al., 2014) | Kenya | Mixture of rural and urban public health facilities | Private locations preferred by the interviewees, primarily homes | To gain a better understanding of the implications of various dimensions of stigma on service use, breastfeeding practices and ART adherence among HIV clients in order to inform the response of integrated HIV services | Integrated HIV and postnatal care services, integrated HIV and family planning services, | HIV positive women who had attended sexual and reproductive health services in study areas | Qualitative in-depth interviews (48). Thematic analysis |
| Adolescents clients’ perceptions of maternity care in KwaZulu-Natal, South Africa (Duggan and Adejumbo, 2012) | South Africa | Mixture of urban public hospital, suburban clinic and rural clinic | Research sites, participant’s home, bedsides in the postnatal ward | To explore adolescent maternity clients’ perceptions of maternity care and to identify important characteristics of an adolescent- friendly maternity service | Antenatal care, postnatal care | Adolescent women aged 15-19 currently using maternity services and comfortable to communicate in English (18) | Focus group discussions (3) and semi-structured interviews (5). Thematic analysis. |
| “What they wanted was to give birth; nothing else”: Barriers to retention in option B+ HIV care among postpartum women in South Africa (Clouse et al., 2014) | South Africa | Urban primary care facility | Private setting at a health centre or via telephone | To identify challenges to retention in care during the postpartum period faced by HIV-positive women initiating ART at a primary health clinic offering Option B+ in South Africa | Option B+ HIV care in postpartum period | Women > 18 years old who were at least 36 weeks pregnant and HIV positive with a mobile phone (interviews then conducted on first postpartum visit) (58) | Mixed methods. Focus group discussions (1), semi-structured interviews (50). Thematic analysis. |
| Postnatal care utilization and local understandings of contagion among HIV-infected and uninfected women in rural. Southern Zambia (Sacks et al., 2016) | Zambia | Hospital within a rural district | Unclear | To examine to effect of HIV-related perceptions on postnatal health care utilization in Choma district, Southern Province, Zambia | Postnatal care | Women recently delivered women (8 women HIV positive, 16 unwilling to disclose status) | In-depth interviews (24). Thematic analysis |
| Psychosocial challenges facing women living with HIV during the perinatal period in rural Uganda (Ashaba et al., 2017) | Uganda | Rural | Unclear | To explore the psychosocial challenges experiences by women living with HIV during pregnancy and postpartum | Pregnancy and postpartum | Women living with HIV who had experienced a pregnancy in the last 2 years | In-depth semi-structured interviews (20)  Thematic analysis. |

**Supplementary Table 3** - Critical Skills Appraisal Programme (2015) summary table

| **CASP Question/ Study** | Was there a clear statement of the aims of the research? | Is qualitative methodology appropriate? | Was the research design appropriate to address the aims of the research? | Was the recruitment strategy appropriate to the aims of the research? | Was the data collected in a way that addressed the research issue | Has the relationship between the researcher and participants been adequately considered? | Have ethical issues been taken into consideration? | Was the data analysis sufficiently rigorous? | Is there a clear statement of findings? |
| --- | --- | --- | --- | --- | --- | --- | --- | --- | --- |
| Amare et al., 2018 | No | Yes | Unclear | Yes | Yes | Unclear | Yes | Yes | Yes |
| Sacks et al., 2017 | Yes | Yes | Unclear | Yes | Yes | No | No | Yes | Yes |
| Kanengoni, Andajani-Sutjahjo and Holroyd, 2019 | Yes | Yes | Unclear | Yes | Yes | No | Unclear | Yes | Yes |
| McMahon et al., 2015 | Yes | Yes | Yes | Yes | Yes | No | Unclear | Yes | Yes |
| Mrisho et al,. 2009 | Yes | Yes | Yes | Unclear | Yes | No | Yes | Unclear | Yes |
| Nabukera et al,. 2006 | Yes | Yes | Unclear | Yes | Unclear | No | Unclear | No | Yes |
| Sialubanje et al., 2014 | Yes | No | No | Yes | Yes | No | Yes | Yes | Yes |
| Clouse et al., 2018 | Yes | No | No | Unclear | Unclear | No | Yes | No | No |
| Zamawe, Masache, Dube, 2015 | Yes | Yes | Yes | Yes | Yes | No | Yes | Yes | Yes |
| Jolly et al., 2019 | Yes | Yes | Yes | Yes | Yes | Unclear | Yes | Unclear | Yes |
| Columbini et al., 2014 | Yes | Yes | Yes | Yes | Yes | No | Yes | Yes | Yes |
| Duggan and Adejumbo, 2012 | Yes | Yes | Yes | Yes | Yes | Yes | Yes | Yes | Yes |
| Clouse et al., 2014 | Yes | Yes | Yes | Yes | Yes | No | Yes | No | Yes |
| Sacks et al., 2016 | Yes | Yes | Yes | Yes | Unclear | No | Yes | No | Yes |
| Ashaba et al., 2017 | Yes | Yes | Yes | Yes | Yes | Unclear | Yes | Yes | Yes |
